# Atomic model validation using the *CCP-EM* software suite

**DOI:** 10.1107/S205979832101278X

**Published:** 2022-01-24

**Authors:** Agnel Praveen Joseph, Mateusz Olek, Sony Malhotra, Peijun Zhang, Kevin Cowtan, Tom Burnley, Martyn D. Winn

**Affiliations:** a Scientific Computing Department, Science and Technology Facilities Council, Didcot, United Kingdom; b Department of Chemistry, University of York, York, United Kingdom; c Electron BioImaging Center, Diamond Light Source, Rutherford Appleton Laboratory, Didcot, United Kingdom

**Keywords:** cryo-EM, model validation, *CCP-EM*, SARS-CoV-2, model geometry

## Abstract

Recently, there has been a dramatic improvement in the quality and quantity of data derived using cryogenic electron microscopy (cryo-EM). This is also associated with a large increase in the number of atomic models built. Although the best resolutions that are achievable are improving, often the local resolution is variable, and a significant majority of data are still resolved at resolutions worse than 3 Å. Model building and refinement is often challenging at these resolutions, and hence atomic model validation becomes even more crucial to identify less reliable regions of the model. Here, a graphical user interface for atomic model validation, implemented in the *CCP-EM* software suite, is presented. It is aimed to develop this into a platform where users can access multiple complementary validation metrics that work across a range of resolutions and obtain a summary of evaluations. Based on the validation estimates from atomic models associated with cryo-EM structures from SARS-CoV-2, it was observed that models typically favor adopting the most common conformations over fitting the observations when compared with the model agreement with data. At low resolutions, the stereochemical quality may be favored over data fit, but care should be taken to ensure that the model agrees with the data in terms of resolvable features. It is demonstrated that further re-refinement can lead to improvement of the agreement with data without the loss of geometric quality. This also highlights the need for improved resolution-dependent weight optimization in model refinement and an effective test for overfitting that would help to guide the refinement process.

## Abstract 2

Utilities for atomic model validation in the *CCP-EM* software suite are discussed. An assessment of SARS-CoV-2 structures reflects a strong bias in model refinement towards geometric restraints when compared with agreement with the data.

## 1. Introduction

Over the last decade, there has been a rapid increase in the number of structures solved using cryogenic electron microscopy (cryo-EM; Callaway, 2020 ▸; Subramaniam, 2019 ▸; Kühlbrandt, 2014 ▸). The resolution of cryo-EM reconstructions has also improved significantly, thanks to technological advances in sample imaging and software for map reconstruction. Currently, the best resolution achieved is 1.15 Å (Yip *et al.*, 2020 ▸) and efforts are ongoing to determine cryo-EM reconstructions at atomic resolutions (Nakane *et al.*, 2020 ▸). Nevertheless, nearly 40% of all reconstructions deposited in the Electron Microscopy Data Bank (EMDB; Patwardhan, 2017 ▸) are in the resolution range 3–5 Å and about 48% are at worse than 5 Å.

The need for cryo-EM map and model validation has been recognized in recent years (Afonine *et al.*, 2018 ▸; Rosenthal & Rubinstein, 2015 ▸; Lawson *et al.*, 2021 ▸), and the EMDR (EMDataResource) map and model challenges (Lawson *et al.*, 2021 ▸; Lawson & Chiu, 2018 ▸) have played a very useful role in comparing existing validation metrics, identifying new requirements and providing data sets for further developments. EM targets have been included in the CASP (Critical Assessment of Protein Structure Prediction) competition since round 13, and the community is invited to submit atomic models for cryo-EM targets (Kryshtafovych *et al.*, 2019 ▸).

Atomic model assessment and validation span different aspects, including model geometry, fit to data, tests for overfitting and model bias. Approaches that evaluate stereochemical properties of the atomic model, such as *MolProbity* (Williams, Headd *et al.*, 2018 ▸) and *CaBLAM* (Prisant *et al.*, 2020 ▸), *PROCHECK* (Laskowski *et al.*, 1993 ▸) and *WHAT_CHECK* (Hooft *et al.*, 1996 ▸), aim to detect potential issues with the geometry of the model by assessing stereochemical properties and comparison with expected standards. Outliers should ideally be fixed where possible prior to automated model refinement (Richardson *et al.*, 2018 ▸). The Ramachandran *Z*-score (Sobolev *et al.*, 2020 ▸; Hooft *et al.*, 1997 ▸) is very useful for detecting an ‘unusual’ φ/ψ dihedral distribution in the model, which is often caused by refinement approaches that overfit the backbone φ/ψ angles to the centroid of allowed Ramachandran space. Another set of methods evaluate each residue in the atomic model based on the local structural neighborhood (Eisenberg *et al.*, 1997 ▸; Sippl, 1993 ▸), which is especially useful to detect errors in the sequence register.

It is also crucial to assess the quality of modeled interfaces between subunits in the cryo-EM-derived assemblies, as they often involve inter-subunit steric clashes, loose interface packing *etc.* (Malhotra *et al.*, 2021 ▸). A recently published score, the Protein Interface score (PI-score), is a metric which can help to distinguish ‘native-like’ interfaces at low-to-intermediate resolution (Malhotra *et al.*, 2021 ▸).

The most common metric used to quantify agreement of the atomic model with the cryo-EM map is the cross-correlation coefficient (CCC; Volkmann & Hanein, 1999 ▸; Roseman, 2000 ▸; Rossmann, 2000 ▸). In Fourier space, the correlation calculated in each resolution shell (Fourier shell correlation; FSC) reflects the agreement of features at each resolution (Brown *et al.*, 2015 ▸). Several other metrics have been tested (Afonine *et al.*, 2018 ▸; Joseph *et al.*, 2017 ▸; Ramírez-Aportela *et al.*, 2021 ▸) and some were found to perform better than others in different resolution ranges and at different degrees of overlap (Joseph *et al.*, 2017 ▸). With data resolution becoming better, multiple methods have been developed to evaluate the agreement with the map at the residue level. The local agreement is either quantified as the real-space CCC in the *Phenix* local CCC (Afonine *et al.*, 2018 ▸), the Manders’ overlap coefficient in SMOC (Joseph *et al.*, 2016 ▸) or a score of atomic resolvability in map *Q* (Pintilie *et al.*, 2020 ▸). The absolute values of most of these metrics vary with the map resolution (Lawson *et al.*, 2021 ▸). The recently introduced FSC-Q score applies normalization to the local FSC to account for local resolution variation (Ramírez-Aportela *et al.*, 2021 ▸).

Another set of metrics evaluate whether atoms in the model are positioned within the molecular contour of the map. The atom-inclusion score (Lagerstedt *et al.*, 2013 ▸) implemented as part of the EMDB validation analysis pages identifies residues that are outside the author-recommended contour of the map. More recently, we developed a tool for assessing the backbone atom positions in the map (Olek & Joseph, 2021 ▸) based on the false-discovery rate-control approach for segregating background noise from molecular volume at a range of resolutions (Beckers *et al.*, 2019 ▸).

Some of the metrics used for model assessment are intrinsically optimized by automated model-refinement approaches, and hence the use of multiple and/or independent metrics is recommended for validation purposes. Atomic model-building and refinement approaches aim to maximize the agreement with map data while satisfying restraints on geometry (Afonine *et al.*, 2018 ▸; Nicholls *et al.*, 2018 ▸). Relative weights for geometry and fit to data are often estimated automatically depending on the data quality. Ideally, the estimated weights are expected to result in an optimal fit to data without overfitting and distorting geometry. Here, we assess the stereochemical quality and fit to data of the deposited models in order to better understand the effect of weights estimated in refinement.

Overfitting is an important factor to consider while trying to optimize the model fit to map. Over the years, several approaches for cross-validation have been proposed to detect overfitting (DiMaio *et al.*, 2013 ▸; Falkner & Schröder, 2013 ▸; Cossio, 2020 ▸; Brown *et al.*, 2015 ▸). However, the requirement for a sufficiently large independent data set has been the primary factor limiting the development of a standardized cross-validation approach, as *R*_free_ is for X-ray crystallography (Brünger, 1992 ▸).

Here, we describe a user-friendly graphical interface which aims to integrate multiple tools for validation that are complementary and/or work on different resolution ranges. The interface is provided as a task within the *Collaborative Computational Project for Electron cryo-Microscopy* (*CCP-EM*) software suite (Burnley *et al.*, 2017 ▸). We also discuss trends from the validation of atomic models determined from SARS-CoV-2 and demonstrate the importance of model agreement with data with the help of a few examples.

## 2. Atomic model-validation task in *CCP-EM*

**Figure 1:**
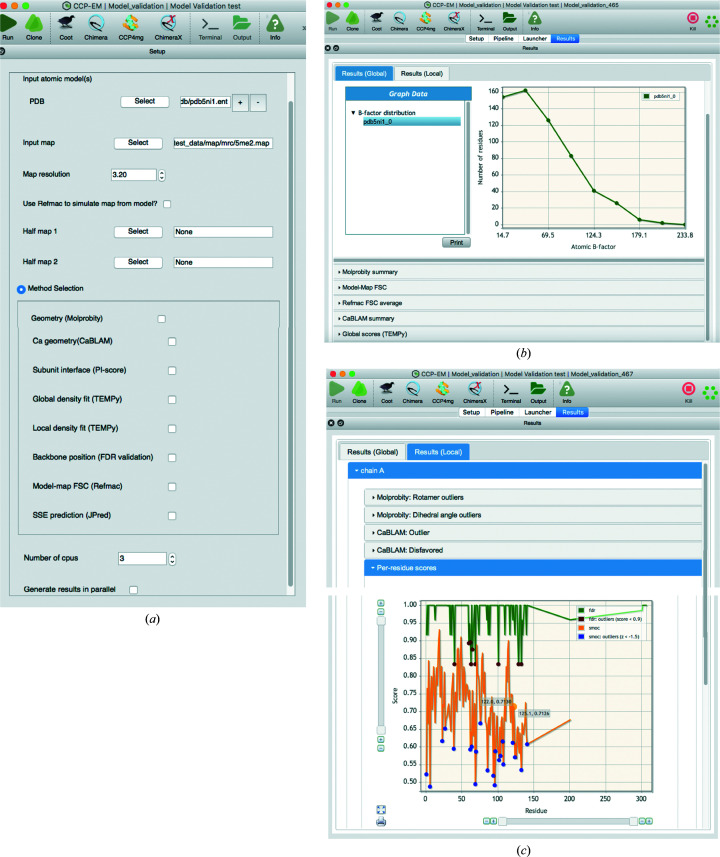
Model-validation task (Validation:model) interface in the *CCP-EM* software suite. The task can be accessed from the list of tasks in the main *CCP-EM* window. The figure shows the ‘Setup’ and ‘Results’ tabs of the interface for calculations on the atomic model of hemoglobin (PDB entry 5ni1) derived from a cryo-EM map at 3.2 Å resolution (Khoshouei *et al.*, 2017 ▸). (*a*) The input setup page lists all input and parameter requirements. Users can choose a selection of assessment tools listed under ‘Method Selection’. (*b*) Global results tab under ‘Results’ showing a list of sections with global statistics returned by different tools. The atomic *B*-factor distribution plot is highlighted. (*c*) Local results tab under ‘Results’ showing a list of sections with outlier details returned by different tools. A per-residue plot of SMOC and FDR-backbone scores is provided under ‘Per-residue scores’. The outlier positions are marked in different colors in this plot.

The *CCP-EM* software suite incorporates a range of functionalities for structure solution under different user interfaces. The atomic model-validation interface (Validation: model) in *CCP-EM* currently integrates multiple tools and metrics that evaluate the geometry of the model and the fit to data. The minimal input for the interface is an atomic model(s) in PDB or mmCIF format that needs to be evaluated for geometry. Computation of scores that quantify the fit to data requires a map and its global resolution as additional inputs (Fig. 1 ▸ *a*). Before calculating the fit-to-data scores, ensure that the model coordinates align with the map grid. Tools for assessing the map quality are not included in this task, but can be found elsewhere in the *CCP-EM* software suite.

The fit-to-data metrics that are part of the task (see below) are sensitive to one or more of the map-processing techniques, including sharpening, filtering, denoising and masking. For a well fitted model, reducing the noise and artifacts (for example from tight masks or over-sharpening) while preserving the signal should ideally improve the fit-to-data scores. Density-modification approaches have been shown to help with refinement and to improve the agreement between the model and map (Terwilliger *et al.*, 2020 ▸; Sanchez-Garcia *et al.*, 2021 ▸; Jakobi *et al.*, 2017 ▸). When using post-processed maps as input, we recommend a careful inspection of the map prior to model validation. Comparison with scores calculated against the raw map might also help to understand any significant effects of post-processing. To compute the FDR-backbone score (see Section 2.2.2), an unmasked input map is required.

The user interface includes graphical tabs allowing access to input parameters, program logs, output files and results. Under the ‘Results’ tab the results are presented for both global and local assessment of the model (Fig. 1 ▸ *b*). It is important to ensure that the atomic *B* factors of the model are refined, as the features of the calculated map are affected by the atomic *B* factors. Hence, the scores may vary significantly depending on whether or not the model *B* factors are refined. A plot of the atomic *B*-factor distribution is provided in the output, and a warning message is displayed when multiple peaks are detected in the distribution. Multiple peaks might indicate partial occupancies or large domain motions, but can also point to inconsistencies in the atomic *B*-factor refinement. The theoretical map calculation from the map is performed using *REFMAC*5 (Nicholls *et al.*, 2018 ▸) by default. If it is turned off, then TEMPy global scores use a more simplistic Gaussian approximation of atoms for map calculation. More systematic approaches for evaluating atomic *B*-factor distributions have recently been developed (Masmaliyeva *et al.*, 2020 ▸) and we plan to integrate such approaches in the future.

The validation tools that are currently part of the validation interface are listed in Sections 2.1 and 2.2.

### 2.1. Model geometry

*MolProbity* (Williams, Headd *et al.*, 2018 ▸) provides statistics on the quality of bonds, angles and dihedrals and serious atomic clashes. Outliers are detected by comparison to standard or expected distributions of geometric parameters. Outlier types include Ramachandran map outliers, rotamer outliers, serious clashes (clashscore), C^β^ deviations, *cis*-peptides and bond-length/angle and dihedral outliers. It is important to ensure that the outliers in the model are justified by the data. The outliers, when present, are often very relevant in terms of the structure stabilization and/or function of the protein.

*MolProbity* reports a single score (*MolProbity* score) which is a weighted combination of clashscore and the percentage of residues in the favorable region of the Ramachandran plot and rotamer outliers. Lower values of the *MolProbity* score reflect better geometry. For crystal structures, a score lower than the crystallographic resolution suggests that the model is better than other structures at this resolution (±0.25 Å) on average. Percentiles associated with clashscore and the *MolProbity* score are also provided to place the model relative to other structures in this resolution range. For a correct interpretation of the percentiles, it is important to ensure that the PDB file header holds the information on data resolution.

*CaBLAM* (Prisant *et al.*, 2020 ▸; Richardson *et al.*, 2018 ▸; Williams, Richardson *et al.*, 2018 ▸) provides statistics on backbone quality based on pseudo-dihedrals consisting of consecutive C^α^ atoms and peptide carbonyls. Outliers are detected based on the position in the pseudo-dihedral space formed by the distribution observed in structures at similar resolutions. In general, a model is expected to have less than 5% *CaBLAM* outliers. *CaBLAM* is particularly useful for lower resolutions, and it is less prone to being refined against.

PI-score (Malhotra *et al.*, 2021 ▸) is a metric that evaluates subunit interfaces in the atomic model and is map-independent. The method computes the PI-score for all of the interfaces present in the input structure and detects potential outliers based on a score cutoff of −0.5 (as recommended by the authors). The chains forming the interfaces identified as outliers are listed in a table under the ‘Global’ results tab. The residues associated with the interface identified as an outlier are also provided as a table under the ‘Local’ outlier tab. Implementation of this score is included in the latest *CCP-EM* nightly release.

Both global and local (outliers) statistics from *MolProbity* and *CaBLAM* are included in the Results.

### 2.2. Fit to map

An atomic model is expected to provide the best representation of experimental data and any interpretations based on the atomic model are also supported by the data. Hence, it is very important to assess the agreement of the model with the data. The model-validation interface in *CCP-EM* integrates approaches that provide both global and local evaluation of the structures.

#### 2.2.1. Global fit to map

*REFMAC*5 (Murshudov *et al.*, 2011 ▸) is used to calculate the model–map Fourier shell correlation (FSC) between a theoretical map calculated from the atomic model and the experimental map (Figs. 1 ▸ *b* and 3*c*). An FSCavg score is derived from the model–map FSC curve by calculating an average of the FSC weighted by the number of structure factors in each shell (Brown *et al.*, 2015 ▸) up to the reported resolution limit. Although higher FSCavg values reflect a better fit, it is necessary to check whether the model starts overfitting to noise. Brown *et al.* (2015 ▸) proposed an approach to estimate model overfitting by comparing model–map FSCs calculated on half maps. The validation interface supports the input of other independent maps for comparing the fit-to-data scores. This is useful as a test for overfitting if the refinement is carried out in one (half) map and an independent additional map input (for example the other half map) can be used to compare the model-validation scores.

*TEMPy* (Cragnolini *et al.*, 2021 ▸; Farabella *et al.*, 2015 ▸) is used to calculate the extent of model–map overlap (OV), real-space cross-correlation coefficient (CCC) and mutual information (MI) scores (Joseph *et al.*, 2017 ▸; Figs. 1 ▸ *b* and 3*d*). A map contour threshold is applied prior to computation of these scores. Users can provide a threshold for contouring the map, which is recommended. The contour level should ideally cover the molecular volume or mask out the background. The choice of contour level is often subjective and for the maps deposited in the EMDB authors often provide a ‘recommended contour level’. The ‘Confidence map’ tool in the *CCP-EM* software package can be used to automatically identify voxels covering the molecular volume (Beckers *et al.*, 2019 ▸). By default, a level corresponding to 1.5σ from the background peak is used as the contour threshold. When three or more models are supplied as input, combined scores that integrate both CCC and MI with extent of overlap (CCC_OV and MI_OV; described in Joseph *et al.*, 2017 ▸) are also calculated. These scores make use of the score distribution to rescale the individual scores before combining them; hence, the calculation requires more than two models.

#### 2.2.2. Local fit to map

*TEMPy* is also used to calculate a segment-based Manders’ overlap coefficient (SMOC) score that quantifies the per-residue agreement between a theoretical map derived from the atomic model and the experimental map (Joseph *et al.*, 2016 ▸). To identify outliers or potential misfits, we compute a *Z*-score for each residue relative to the local neighborhood (residues within 12 Å). Residues associated with *Z*-scores of <−1.5 are identified as potential outliers.

The confidence-map tool developed by Beckers *et al.* (2019 ▸) is used to assess the coordinate positions of backbone atoms in the model (Olek & Joseph, 2021 ▸). The backbone validation score reflects whether the residue backbone is traced in the molecular volume or background noise. An unmasked map is required as input for the successful computation of this score. This score was demonstrated to be complementary to other existing scores that quantify model agreement with maps. Residues associated with scores lower than 0.9 usually require attention and are designated as outliers. Implementation of this score is included in the latest *CCP-EM* nightly release. Supplementary Fig. S2 shows an example from the EMDB Model Challenge 2019 (Lawson *et al.*, 2021 ▸) where the FDR-backbone score detects potential issues with the backbone trace.

*JPred*4 is used to predict the secondary structure from the sequence of the protein chain (Drozdetskiy *et al.*, 2015 ▸). The high-confidence predictions for helical and strand conformations are then compared against the secondary-structure type observed in the atomic model as assigned using *DSSP* (Kabsch & Sander, 1983 ▸). Mismatches are reported as outliers, although it is important to note that the accuracy of the prediction from sequence is only about 80–85% (Buchan & Jones, 2019 ▸; Drozdetskiy *et al.*, 2015 ▸; Yang *et al.*, 2018 ▸). Hence, we recommend prioritizing cases where there is low agreement with the map (outliers based on fit-to-data metrics) and a mismatch with the secondary-structure prediction from the sequence. In this case, it is possible that the modeled secondary structure is incorrect and needs to be fixed.

In the *CCP-EM* model-validation interface, per-residue SMOC and FDR-backbone scores are provided as a plot under the local tab, and outliers identified are also highlighted (Fig. 1 ▸ *c*). Table 1 ▸ gives a summary of tools currently accessible from the validation task.

### 2.3. Outlier clusters

**Figure 2:**
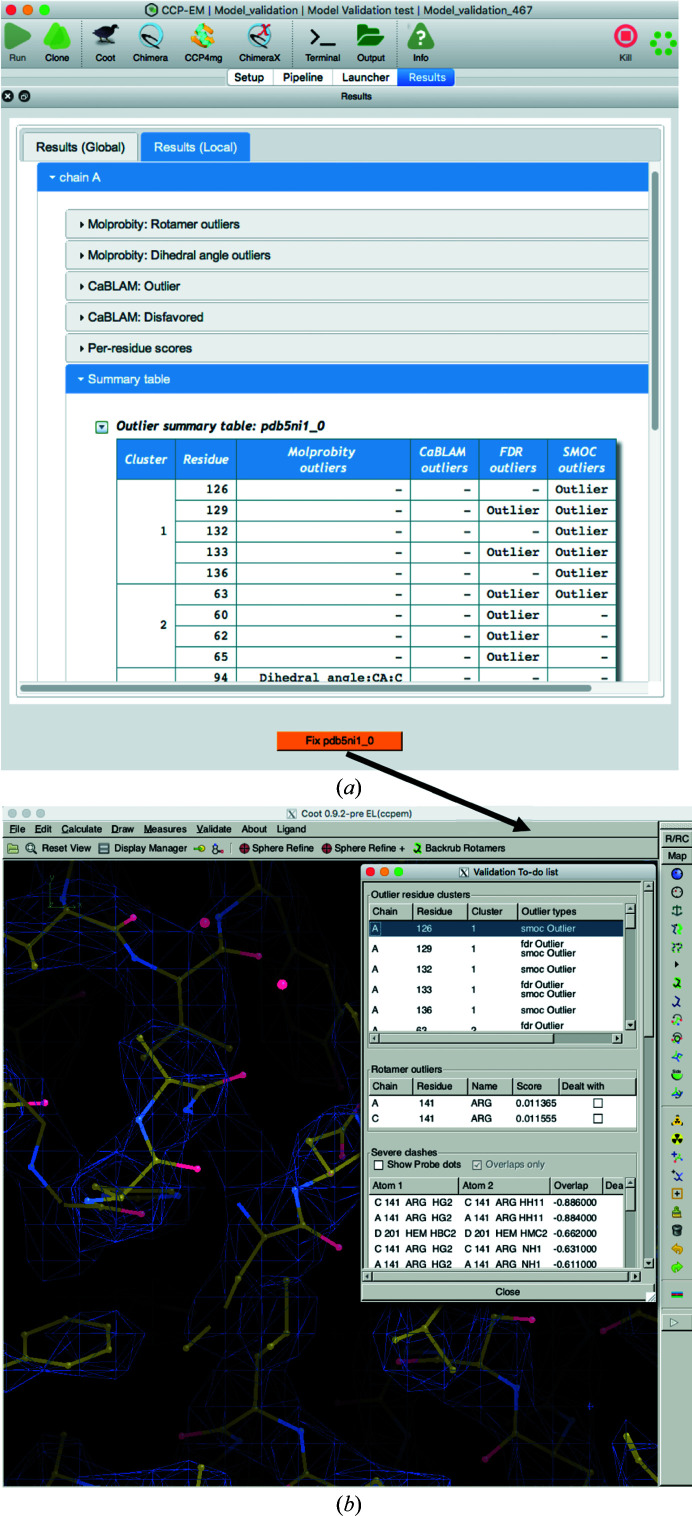
Summary table with outlier clusters. (*a*) Local results of the *CCP-EM* model-validation task, highlighting the table with outliers clustered in space. The clusters are ranked by size and the last row includes all residues that are not part of any cluster. (*b*) Clicking the orange button at the bottom of the local results page opens the map and the atomic model in *Coot* and a window with a list of outliers that need fixing, again ordered by clusters.

Often, atomic models derived from cryo-EM data are associated with a large number of outliers that arise from different metrics and are distributed across the structure. To highlight specific structural regions with the most serious issues, we cluster outliers with C^α^ atoms that are within 7 Å of each other and provide a summary table with a list of clusters ordered by cluster size (a validation ‘to-do’ list). Users can proceed with examining the outliers in *Coot* (Emsley *et al.*, 2010 ▸) by clicking the button at the bottom of the Results page (Fig. 2 ▸ *a*). This opens the model and map in *Coot* along with the list of outlier clusters (Fig. 2 ▸ *b*). The issues can be fixed interactively in *Coot* and flagged as complete when each residue is fixed.

Access to *MolProbity*, *CaBLAM *and *REFMAC*5 via the *CCP-EM* interface currently requires installation of the *CCP*4 software suite (Winn *et al.*, 2011 ▸).

## 3. Assessment of cryo-EM structures from SARS-CoV-2

### 3.1. Assessment using the *CCP-EM* model-validation task

A set of 298 models derived from cryo-EM structures from SARS-CoV-2 were available in the PDB at the end of March 2021. We filtered out models for which the coordinates do not overlap with the map, reflecting potential issues with the relative positioning of the map (grid origin) and model coordinates in space (20/298). This resulted in a set of 278 models that were assessed using the validation suite.

**Figure 3:**
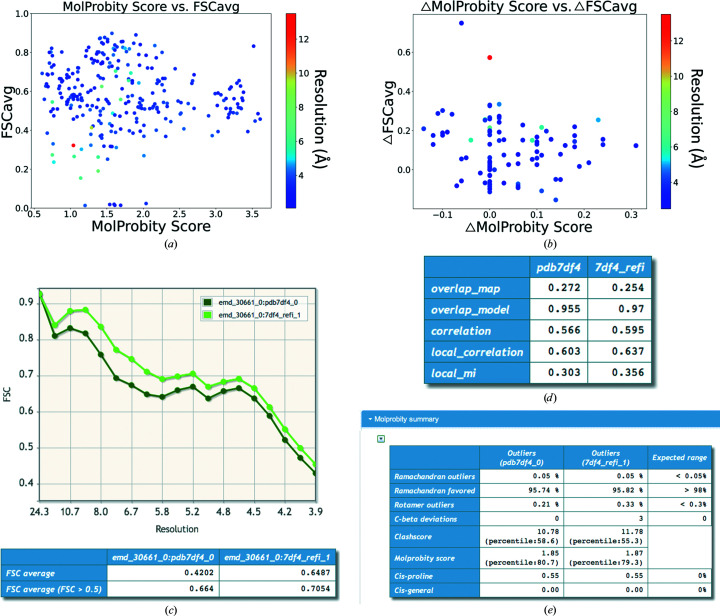
Trends of model geometry versus fit to data for 252 deposited SARS-CoV-2 models. In (*a*) and (*b*), the points are colored based on the resolution of the map, and the color bar on the right shows the colors with respect to resolution. (*a*) Distribution of *MolProbity* scores versus FSCavg. (*b*) Plot of difference in scores (refined − initial) for a random set of 100 models that were re-refined: (*c*) model–map FSC curves and FSCavg calculated using *REFMAC*5, (*d*) table with different scores on fit to data calculated using *TEMPy* and (*e*) comparison of *MolProbity* statistics.

75% of the data set corresponded to models derived from maps resolved at worse than 3.0 Å resolution. 44% of the models had *MolProbity* scores better than 1.5 (Fig. 3 ▸ *a*) and 36% had no Ramachandran outliers. A *MolProbity* score of 1.5 reflects that the model is of comparable geometric quality to structures resolved at a crystallographic resolution of 1.5 Å (Chen *et al.*, 2010 ▸). However, in practice, with refinement approaches becoming better and the addition of new models, this correspondence may not be accurate. Nevertheless, the score gives an indication of the quality of the model, which is expected to vary with the data quality. The median of *MolProbity* scores associated with structures better than 3.0 Å resolution is 1.53 and the medians of the FSCavg and CCC scores are 0.62 and 0.87, respectively. On the other hand, the median of the *MolProbity* scores associated with structures worse than 4.0 Å resolution is 1.54 and the medians of the FSCavg and CCC scores are 0.50 and 0.47, respectively.

Clearly, the geometry is heavily restrained by refinement approaches at low resolutions and the (implicit) inclusion of Ramachandran restraints in refinement might have contributed to the absence of Ramachandran outliers in a significant number of models. On the other hand, fit-to-data scores are often poor, particularly for lower resolution structures. To check whether the fit to data can be improved further, we randomly selected 100 models from the data set. The models were then subjected to 20 cycles of local refinement using the *REFMAC*5 implementation in *CCP-EM* (Burnley *et al.*, 2017 ▸; Nicholls *et al.*, 2018 ▸). In *REFMAC*5, automated weight estimation (keyword: weight auto) identifies a relative weight (geometry restraints versus fit to data) that maintains the target bond r.m.s.d. within 0.01 and 0.02 Å. However, as recommended in the *REFMAC*5 documentation (https://www2.mrc-lmb.cam.ac.uk/groups/murshudov/content/refmac/refmac_keywords.html), the relative weight for the data needs to be optimized further for use with cryo-EM maps. New developments in *REFMAC* (Yamashita *et al.*, 2021 ▸) include better weight estimation within the range 0.2–18.0, depending on the resolution and the ratio of model to map volumes.

From our experience, a relative weight of between 2 and 4 works well at resolutions of 3 Å or worse. In this study, we used a weight of 3 using the ‘weight auto 3’ keyword, which sets a relatively lower starting weight of 3. Note that this weight may not be optimal for all maps in the data set and further interactive refinement and error fixes may be required on a case-by-case basis after automated refinement. Nevertheless, we wanted to check whether the automated refinement helps to improve agreement with the data without a significant decline in geometric quality.

Using this automated protocol, FSCavg improved in 71% of the re-refined models while 20% had a lower FSCavg score. 34% of the models had both a better FSCavg score and the same or an improved *MolProbity* score (Fig. 3 ▸ *b*). Where the *MolProbity* score became worse, the change was not large (less than 0.2 for all but five models), suggesting that the majority of the re-refined models had comparable geometric quality to the respective initial model.

Hence, the fit to data could be further improved in a significant majority of these cases. Figs. 3 ▸(*c*)–3 ▸(*e*) show an example (PDB entry 7df4) where the FSCavg improved from 0.42 to 0.65, with no significant change in the *MolProbity* score. The global statistics from the *CCP-EM* model-validation interface highlight the improvement based on multiple metrics.

### 3.2. Assessment of backbone tracing

We calculated the FDR-backbone scores for the data set of atomic models associated with cryo-EM structures from SARS-CoV-2. As the confidence-map calculation (Beckers *et al.*, 2019 ▸) requires unmasked maps as input, we filtered out maps deposited in EMDB that were post-processed with the application of a mask. The backbone trace of the remaining 199 SARS-CoV-2 models were evaluated using the FDR-backbone score (Olek & Joseph, 2021 ▸). The FDR-backbone score allows us to easily identify and locate potential issues with backbone tracing in the model. Residues with an FDR-backbone score of lower than 0.9 are potentially misplaced and might require additional refinement. The quality of the model can be represented with an overall FDR-backbone metric, which is calculated as the fraction of residues with a score of higher than 0.9.

**Figure 4:**
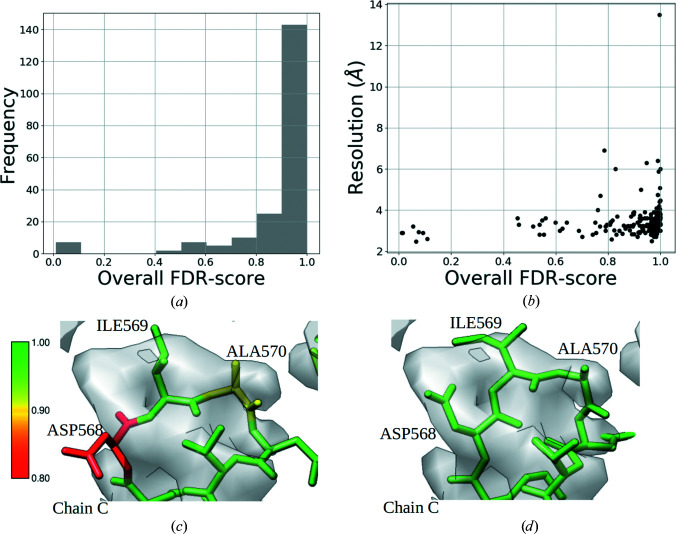
(*a*) Histogram of the overall FDR-backbone metric from the SARS-CoV-2 data set. (*b*) Plot of the overall FDR-backbone metric versus resolution. (*c*) A region of the deposited model (residues 568–571, PDB entry 7c21) colored by the per-residue FDR-backbone scores. (*d*) The re-refined model colored by the per-residue FDR-backbone scores.

Fig. 4 ▸(*a*) shows the distribution of the overall FDR-backbone metric. 143 of 199 models have the backbone atoms correctly placed for at least 90% of the residues. 56 models out of 199 had an overall FDR-backbone score of lower than 0.9. Of the 147 models built from maps with reported resolution 3–4 Å, 37 were associated with an overall FDR-backbone metric of less than 0.9 (Fig. 4 ▸ *b*), reflecting potential issues with the backbone trace. We checked whether further re-refinement could improve the FDR-backbone score. To demonstrate this, we selected a model (PDB entry 7c2l) associated with a low FDR-backbone score of 0.8. A region with several residues with lower FDR-backbone scores (chain *C*, residues 568–571; Fig. 4 ▸ *c*) was re-refined using the real-space refinement tools in *Coot* (*Sphere and Zone Refine*; Emsley *et al.*, 2010 ▸; Fig. 4 ▸ *c*). The interactive refinement resulted in an improved fit of the residues in the map, which was also associated with improved FDR-backbone scores, with most residues having a score of 1.0 (Fig. 4 ▸ *d*).

### 3.3. Assessment of modeled interfaces

We further assessed the quality of protein interfaces in the fitted models for the SARS-CoV-2 cryo-EM structures. The structures were subjected to quality assessment using the Protein-Interface quality score (PI-score; Malhotra *et al.*, 2021 ▸). PI-score is a machine-learning-based score which uses derived features of the interfaces for training. Here, we have retrained the PI-score machine-learning model without using sequence conservation as one of the derived interface features, as we have previously shown that other features such as shape complementarity were ranked much higher than conservation (Malhotra *et al.*, 2021 ▸). The calculation of residue conservation at the interface is a very time-consuming step, as one needs to collect homologs and build a multiple sequence alignment. The model accuracy was not significantly affected when conservation was not used as one of the derived interface features (Supplementary Fig. S3).

**Figure 5:**
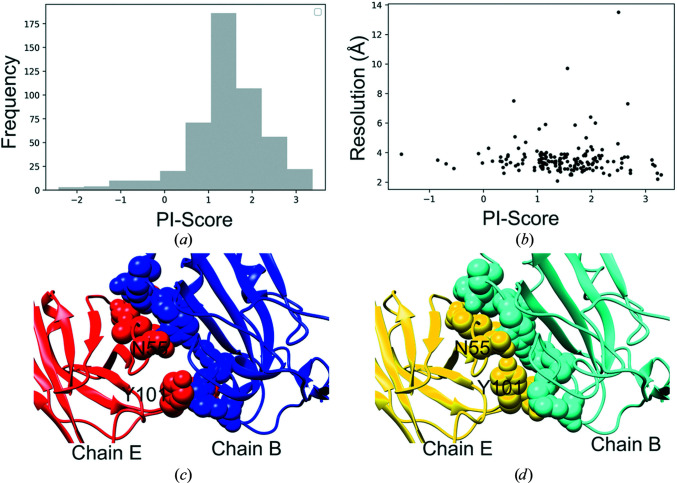
Assessment of modeled interfaces in SARS-CoV2 cryo-EM assemblies. (*a*) Distribution of PI-score for the SARS-CoV-2 assemblies. (*b*) Plot of PI-scores (averaged over all interfaces within a structure) against the resolution of the structures. (*c*) Modeled interface between chain *B* (spike) and chain *E* (heavy chain of the antibody) in one of the open-state structures (PDB entry 7cac; 3.55 Å resolution) which was scored negative. (*d*) Re-refined structures for chains *B* and *E* of PDB entry 7cac, obtained using *Coot* real-space refinement, show improved shape complementarity of the protein–protein interface.

All of the required features to calculate PI-score were successfully calculated for 489 interfaces from 178 SARS-CoV-2 cryo-EM-derived structures. These interfaces were then assessed for their interface quality using PI-score. 94% of the interfaces and even those modeled from low-resolution data scored positive, indicating the good-quality interfaces modeled within the cryo-EM structures (Figs. 5 ▸ *a* and 5 ▸ *b*).

We further investigated one of the interfaces which was scored negative (PDB entry 7cac, chains *B* and *E*, PI-score = −1.2, resolution 3.55 Å). This interface is between the receptor-binding domain of the spike protein (chain *B*) and the antibody heavy chain (chain *E*). The interface was scored low on shape complementarity (*sc* score 0.44; Lawrence & Colman, 1993 ▸) and has clashes at the interface (Fig. 5 ▸ *c*). We further re-refined this structure in *Coot* (*Sphere Refine*), which helped to resolve some of the clashes and improved the shape-complementarity score (to 0.67; Fig. 5 ▸ *d*). Subsequently, the re-refined structure obtained a positive PI-score of 1.57. In this case, using the CCC and SMOC score calculated on interface residues (iSMOC), one cannot distinguish between the deposited structure (CCC = 0.75 and iSMOC = 0.25) and the re-refined structure (CCC = 0.74 and iSMOC = 0.26), whereas the PI-score can help to locate the errors at the interface. Hence, the PI-score provides complementary validation assessment for protein–protein interfaces which can be very helpful in cases such as these.

## 4. Availability and future perspectives

The interface for model validation (Validation:model) is available in the *CCP-EM* software package, which is downloadable from https://www.ccpem.ac.uk/download.php. Based on the assessment of cryo-EM structures from SARS-CoV-2, we observe a clear bias towards model geometry when compared with agreement with data. Although model geometry may be favored at low resolutions due to the low information content associated with the data, care should be taken to ensure agreement with resolvable features in the map. To this end, there is a need for validation tools that evaluate the quality of low-resolution features of a model and their agreement with the map. This is also relevant for tomogram reconstructions and subtomogram averages from cryo-electron tomography. Currently, there is a lack of a robust test for overfitting that will help with the selection of refinement weights (DiMaio *et al.*, 2013 ▸) and optimization of model fit to map. In fact, the models currently available from the PDB might benefit from further re-refinement. In this context, efforts such as *CERES* (Liebschner *et al.*, 2021 ▸) and the extension of *PDB-REDO* (Joosten *et al.*, 2014 ▸) to models derived from cryo-EM will be of increasing importance. As most of the cryo-EM reconstructions suffer from variable local resolution, an overall bias towards model geometry will affect fit in the better resolved areas. To address this, local resolution-dependent weight optimization for model refinement would be a good step forward.

The pipeline underlying the validation task has been used to evaluate all cryo-EM structures from SARS-CoV-2 and the results have been deposited in the public repository maintained by the Coronavirus Structure Task Force (Croll *et al.*, 2021 ▸). In this study, we show examples where validation metrics evaluate different features of the model and highlight associated potential issues. These issues were then fixed using interactive refinement in *Coot*.

The validation task in *CCP-EM* highlights the crucial areas associated with more serious issues by clustering outliers in space. The interface also provides a way to fix the outlier clusters in *Coot*. In the future, we plan to expand the validation task with other validation tools including tools for the validation of nucleic acids and carbohydrates. We also aim to include functionality to recalculate these scores from the *Coot* interface upon fixing the outliers.

## Supplementary Material

Supplementary Figures. DOI: 10.1107/S205979832101278X/qg5004sup1.pdf

## Figures and Tables

**Table 1:** List of tools currently included in the *CCP-EM* model-validation task

Metric(s)	Tool	Evaluation
Ramachandran plot, rotamers, serious clashes, bond lengths, bond angles, other dihedrals	*MolProbity*	Global and local geometry
*CaBLAM*	*MolProbity*	Global and local backbone geometry
Protein Interface score	PI-score	Protein–protein interface quality
Model–map FSC, FSCavg	*REFMAC*5	Global fit to data
CCC, MI, OV, CCC_OV, MI_OV	*TEMPy*	Global fit to data
SMOC	*TEMPy*	Local fit to data
FDR-backbone score	FDR thresholding (confidence map), FDR backbone validation tool	Local backbone trace
Secondary-structure prediction	*JPred*4	Secondary structure (local)
