# Supplementary material for: Atomic model validation using the CCP-EM software suite
Source: Acta Crystallogr D Struct Biol. 2022 Jan 24;78(Pt 2):152–61. doi: 10.1107/S205979832101278X (PMC8805302; doi:10.1107/S205979832101278X)
Supplement: Supplementary file 1 [file d-78-00152-sup1.pdf]

Acta Cryst D STRUCTURAL BIOLOGY

Volume 78 (2022)

Supporting information for article:

Atomic model validation using the *CCP-EM* software suite

Agnel Praveen Joseph, Mateusz Olek, Sony Malhotra, Peijun Zhang, Kevin Cowtan, Tom Burnley and Martyn D. Winn

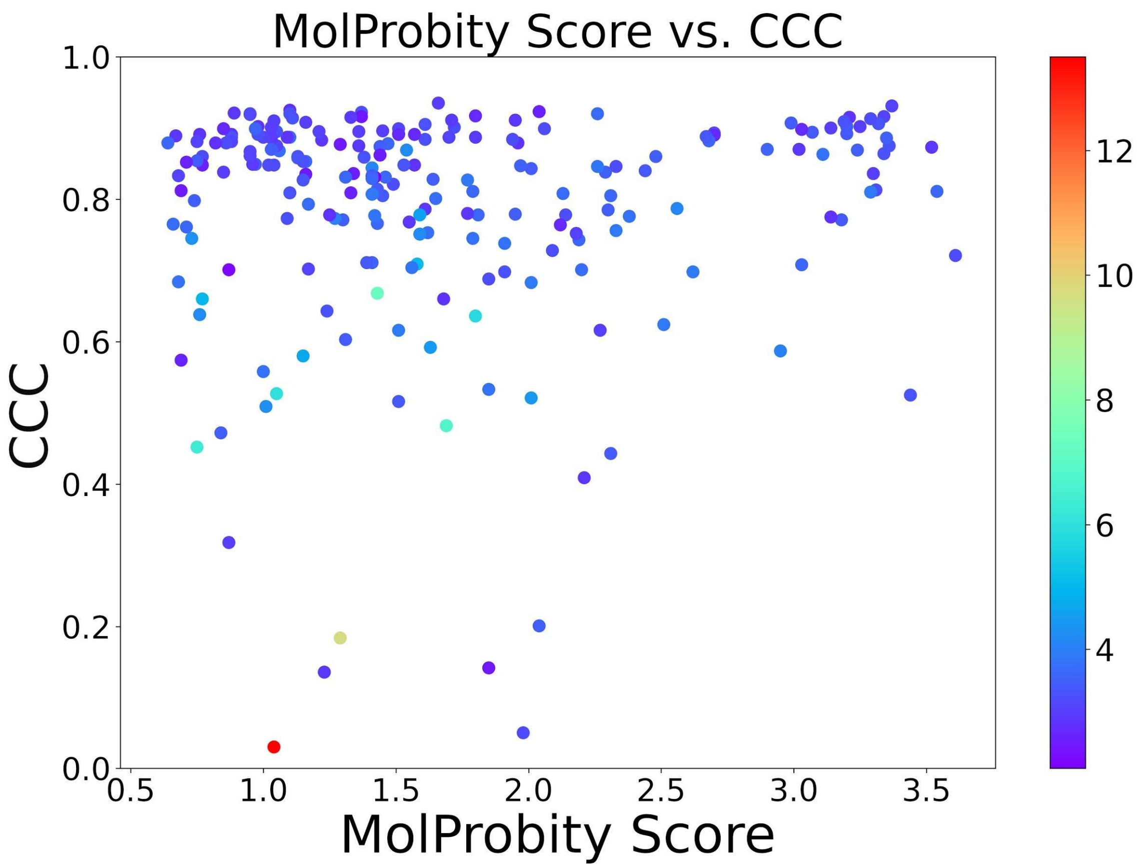

Figure S1 Trends of Molprobity score vs CCC for SARS-CoV-2 models in the dataset. The points are colored according to the resolution of the map, the color-bar on the right shows the color-scale with respect to resolution.

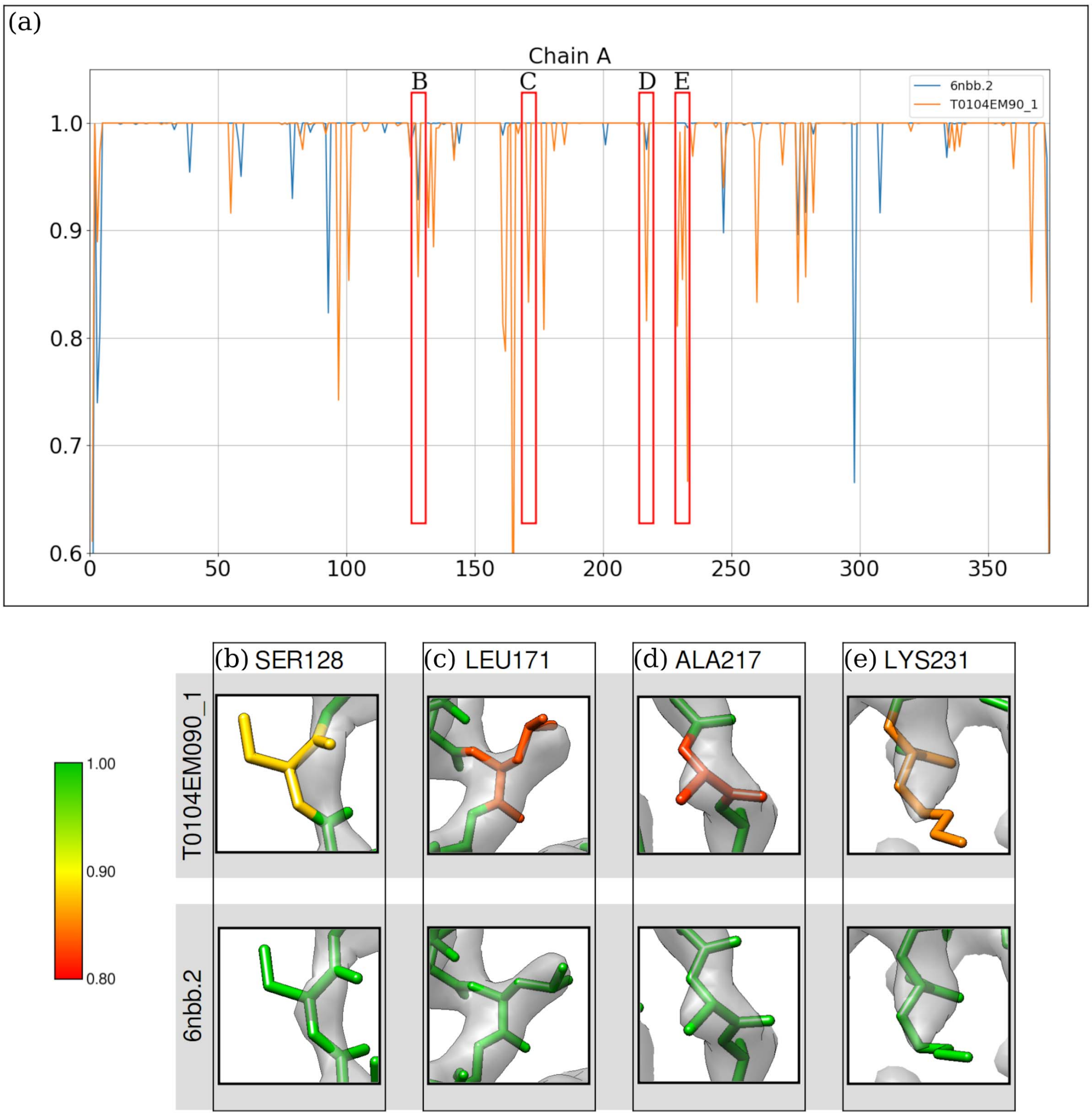

Figure S2 Comparison of FDR backbone scores for the Chain A of the atomic models of alcohol dehydrogenase T0104EM090_1 from the EMDB model challenge and the reference model (PDB ID: 6nbb.2) used in the challenge. (A) The metrics calculated only for the backbone atoms of the Chain A, the red boxes highlight a few of the low-scoring residues selected for comparison in the panels below. For Ser 128 (B), Leu 171 (C), Ala 217 (D) and Lys 231 (E), the panel shows the residue fit in the target map (EMD-0406, grey) displayed at the recommended contour level and rendered in UCSF Chimera. Color bar shows the correspondence with the FDR scores assigned, residues in red have FDR scores around 0.8 or worse, yellow around 0.9 and green around 1.0. The potential issues with the backbone trace detected based on the score is also reflected by a better backbone fit in the reference model associated with FDR-backbone scores of 1.0 (colored in green).

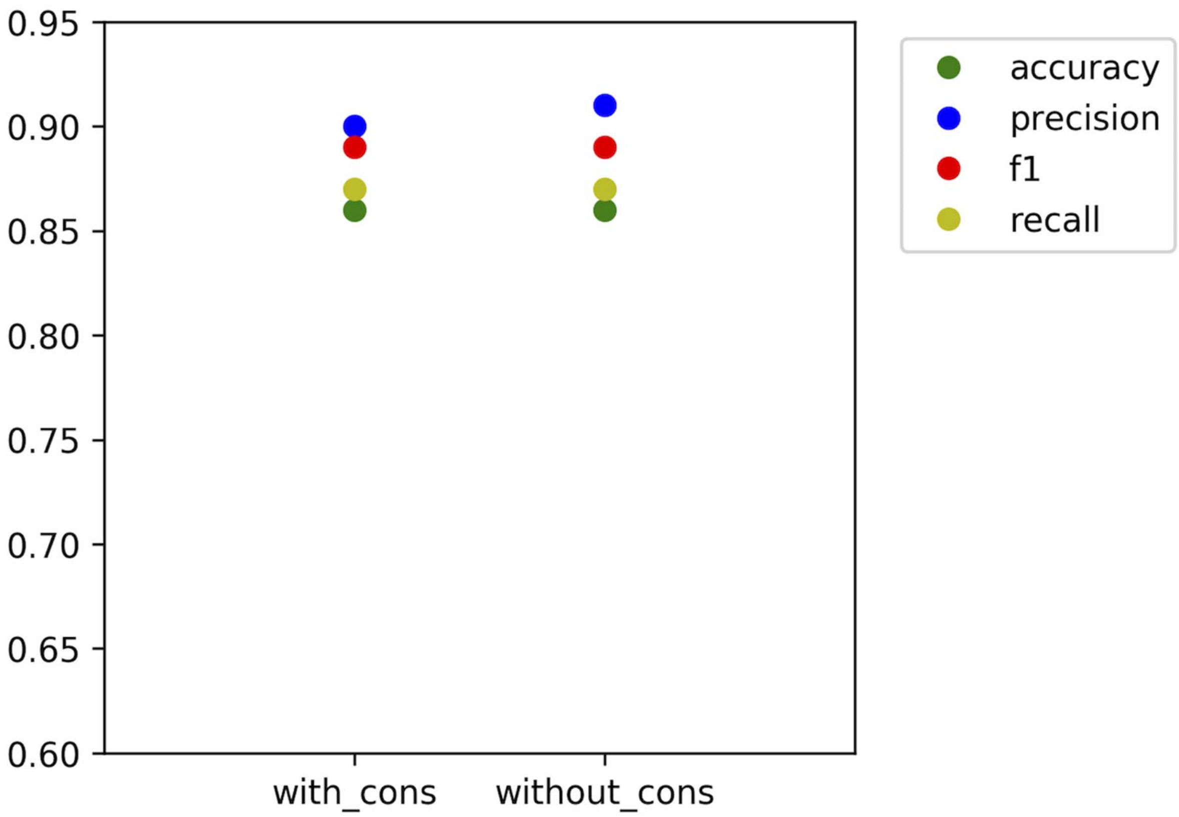

Figure S3 Machine learning model for interface quality. Performance measures for the models when trained with and without conservation as one of interface features.
